# Adherence to the Mediterranean Diet as a Modifiable Risk Factor for Thyroid Nodular Disease and Thyroid Cancer: Results From a Pilot Study

**DOI:** 10.3389/fnut.2022.944200

**Published:** 2022-06-17

**Authors:** Luigi Barrea, Giovanna Muscogiuri, Giulia de Alteriis, Tommaso Porcelli, Claudia Vetrani, Ludovica Verde, Sara Aprano, Francesco Fonderico, Giancarlo Troncone, Annamaria Colao, Silvia Savastano

**Affiliations:** ^1^Dipartimento di Scienze Umanistiche, Università Telematica Pegaso, Naples, Italy; ^2^Endocrinology Unit, Department of Clinical Medicine and Surgery, Centro Italiano per la cura e il Benessere del paziente con Obesità (C.I.B.O), University Medical School of Naples, Naples, Italy; ^3^Unit of Endocrinology, Dipartimento di Medicina Clinica e Chirurgia, Federico II University Medical School of Naples, Naples, Italy; ^4^Cattedra Unesco “Educazione alla salute e allo sviluppo sostenibile”, University of Naples Federico II, Naples, Italy; ^5^Department of Public Health, University of Naples Federico II, Naples, Italy

**Keywords:** Mediterranean diet (MD), thyroid nodular disease, thyroid cancer, FNA, Tir, PREvención con DIeta MEDiterránea (PREDIMED), nutritionist, fine needle aspiration (FNA)

## Abstract

Iodine deficiency is the most important established nutritional risk factor for the development of thyroid nodular disease. Nevertheless, to the best of our knowledge, to date no study focused on the association between the adherence to the Mediterranean diet (MD) and thyroid nodular disease. Adherence to the MD was evaluated using the PREvención con DIetaMEDiterránea (PREDIMED) questionnaire. Physical activity, smoking habits, and anthropometric parameters were studied. PREDIMED was used to evaluate the degree of adherence to the MD. Evaluation of fine needle aspiration cytology of thyroid lesions based on 2013 Italian thyroid cytology classification system. Cytology of thyroid nodules was carried out through sonography-guided fine-needle aspiration and patients were divided into 5 categories: TIR2, TIR3a, TIR3b, TIR4, and TIR5. The study population consisted of 794 subjects (554 females, 69.8%), aged 18–65 years, with BMIs ranging from 19.4 to 55.3 kg/m^2^. Thyroid nodular disease was present in 391 participants (49.2%), and the most frequent cytological categories was TIR2 (18.3 %), followed by a TIR4 (8.9 %). The presence of thyroid nodules was also significantly associated with the lowest adherence to the MD (OR 6.16, *p* < 0.001). Patients with TIR5 had the lower adherence to the MD (2.15 ± 1.12 score) compared to other TIRs (*p* < 0.001). The cytological category with high-risk of malignancy (TIR4/TIR5) was significantly associated with the lowest adherence to the MD (OR 137.55, *p* < 0.001) and PREDIMED score (OR = 0.33, *p* < 0.001, 95% IC = 0.26–0.41, *R*^2^ = 0.462). At multiple regression analysis, PREDIMED score was the main predictor of both the presence of nodules (*p* < 0.001) and the cytological category with high-risk of malignancy (*p* < 0.001). At ROC analysis PREDIMED score ≤ 5 and ≤ 4 (*p* = 0.001) were the values that predicted the presence of thyroid nodular disease and cytological category with high-risk of malignancy, respectively. In conclusion, our study demonstrated that the low adherence to the MD is associated with the presence of thyroid nodular disease and in particular with those at high-risk of malignancy.

## Introduction

Iodine deficiency represents the most important established nutritional risk factor for the development of thyroid nodular diseases (1). Furthermore, iodine intake is also a risk factor for thyroid cancer (2), with a U-shaped association between iodine consumption and thyroid diseases, indicating that either high or a low iodine intake will lead to thyroid diseases (3). A number of different modifying and non-modifying factors could account for the increased incidence of benign/malign thyroid nodular disease (4). In particular, unhealthy dietary pattern could contribute to the pathogenesis of thyroid nodular disease, although there are not sufficient studies to establish if this was due to a direct effect of unhealthy nutrition or mediated by obesity, insulin resistance, and inflammation that are usually a consequence of unhealthy nutrition (1).

Along with obesity, the incidence of both benign and malign thyroid nodular disease is increasing worldwide (5). However, the association between obesity epidemic and benign thyroid nodular disease or thyroid cancer is still debated (6, 7). In particular, there is conflicting evidence on the association between obesity and differentiate thyroid cancer, the most common endocrine malignancy, and differentiate thyroid cancer aggressiveness (8).

In this regard, the inflammatory potential and insulin resistance related to unhealthy dietary pattern are well-known (9, 10). In particular, inflammation (11) and insulin resistance (12, 13) have gained growing interest in promoting thyroid cell hyperplasia, thus facilitating the prevalence and malignant growth in thyroid nodular disease. To increase the knowledge on nutritional factors might be of crucial importance for organizing thyroid nodular disease prevention strategies (14–16).

Indeed, rarely the record of the consumption of some single foods, including red meat, and nut intake, alcohol drinking, has been included in the clinical evaluation of thyroid nodular disease (17, 18). In particular, Yao et al. reported that red meat consumption was an independent risk factor of thyroid nodular disease, while nut consumption was an independent protective factor (18).

Some nutritional patterns, together with excessive weight, seem to also play a relevant role in thyroid cancer carcinogenesis, although a clear association between dietary factors and thyroid cancer has not been defined so far (1, 19). In particular, different single foods have been reported to have protective effects on thyroid cancer risk, including fish (1, 20), vegetables (21), mainly cruciferous intake (1, 22), fruit (21). Nevertheless, the studies were conducted only in Western country population (23), and their results were globally inconsistent (24).

In addition, diet is a complex and synergistic combination of single foods, which are not individually consumed (25). For this reason, in free-living populations, it is challenging, to separate the effect of single foods from others (26). In this context, the evaluation of a healthful dietary pattern, as Mediterranean diet (MD), in patients with thyroid nodular disease and grouped according to their cytological classification has been never investigated previously.

The Mediterranean-style dietary pattern has demonstrated to represent as the dietary pattern that globally reflects the characteristics of a healthy diet effective in tackling obesity epidemic (27) and obesity-related consequences, including cardiovascular diseases (28), and cancer (29). The anti-inflammatory and anti-oxidant properties exerted by the MD is widely recognized (30, 31).

According to current guidelines, management of thyroid nodular disease is primarily based on morphologic classification of the cytological samples obtained by fine-needle aspiration (FNA) complemented by clinical history, imaging findings, and molecular markers test results (32–34). Nevertheless, the nutritional aspects of patients with thyroid nodular disease have generally been unexplored.

A primary aim of this study was to investigate the association between adherence to the MD and presence of thyroid nodular disease in a large cohort of adult population, providing a detailed information about single MD food items in patients with thyroid nodule presence. As second aim, in the subset of the study participants who have undergone FNA, we have evaluated adherence to the MD according to the cytological classification of thyroid nodules.

## Materials and Methods

This monocentric study was carried out in patients attending the Obesity Unit (C.I.B.O. and EASO center) at the Unit of Endocrinology, Clinical Medicine and Surgery Department, University “Federico II” of Naples from January 2015 to January 2021. Federico II Ethical Committee approved this study protocol (n. 239/11). This study was carried out in accordance with the Declaration of Helsinki (Code of Ethics of the World Medical Association) for experiments that involved humans. Part of the participants were also recruited during the OPERA prevention project for details see “The opera prevention project” (35). All participants were informed about the study design and aim and gave informed consent. This study included 794 participants, coming from the same geographical area around Naples metropolitan area, Campania, Italy.

### Thyroid Assessments

In all participants, thyroid nodular disease was evaluated at the Endocrinology Unit of Federico II Hospital (Naples, Italy). In the subset of the study participants with thyroid nodular disease, the cytological samples obtained by FNA, have been analyzed of the Pathology Department, according to the Italian consensus for the classification and reporting of thyroid cytology (36). Based on this classification, patients were divided into 5 categories: TIR2, non-malignant/benign; TIR3a, low-risk indeterminate lesion; TIR3b, high-risk indeterminate lesion; TIR4, suspicious of malignancy, and TIR5, malignant. In particular, in this study we grouped the patients into two different risk categories, low risk or uncertain risk of malignancy (TIR2, TIR3a, and TIR3b) and high risk of malignancy (TIR4/TIR5). Thyroid nodular disease with TIR1 cytology were excluded from this study.

### Physical Activity and Smoking Habits

Physical activity levels and smoking habits were evaluated in all participants through the administration of a standard questionnaire, as already reported in other studies (37, 38). Subjects who practiced at least 30 min of daily aerobic physical activity were classified as active (YES). Similarly, when participants smoking at least one cigarette per day were classified as current smokers (YES), as we reported earlier (39, 40).

### Anthropometric Measurements

All anthropometric measurements, including weight and height were done after an overnight fast, between 9 and 10 a.m. in all participants who wore light clothing and were without shoes. In particular, a wall-mounted stadiometer to the nearest 1 cm and a calibrated balance beam scale was used to the nearest 50 g (Seca 711; Seca, Hamburg, Germany) were used for height and weight measurement, respectively. Body mass index (BMI) was calculated by weight (kg) and height squared (m^2^) and subjects were classified into five BMI classes: normal weight, overweight, grade I obesity, grade II obesity, and grade III obesity (BMI: 18.5–24.9, 25.0–29.9, 30.0–34.9, 35.0–39.9, and ≥40.0 kg/m^2^, respectively), as previously reported (41) and in accordance with the WHO's criteria WHO (42).

### Adherence to the MD

A previously validated of 14-item questionnaire PREvention with MEDiterranean Diet (PREDIMED) (43) questionnaire, was assessed for evaluated adherence to the MD (43), as we reported earlier (44–46). This questionnaire was performed by a certified clinical nutritionist through a face-to-face interview with all the enrolled participants. Scores of zero (No) and one (Yes), were given for questions of PREDIMED questionnaire. From the sum of the 14 questions of the PREDIMED questionnaire, PREDIMED score was calculated. From the totalized PREDIMED score values, participants we divided into three categories of adherence to the MD: highest adherence, average adherence, and lowest adherence to the MD (PREDIMED score ≥10, 6–9, and 0–5, respectively) (44–46).

### Statistical Analysis

Kolmogorov-Smirnov test was used to evaluate the data distribution and the abnormal data were normalized by logarithm. Skewed variables were back-transformed for presentation in tables and figures. The chi-square (χ^2^) test was used to determine the statistically significant differences in the frequency distribution, including sex, lifestyle characteristics, anthropometric measurements, nutritional and thyroid parameters. The differences in continuous variables between nodules presence/absence were compared using the Student's unpaired *t*-test, while the differences in multiple groups (cytology categories based on 2013 Italian thyroid cytology classification system) were analyzed by ANOVA test followed by the Bonferroni *post-hoc* test. A multinomial logistic regression model, odds ratio (OR), *p*-value, 95% interval confidence (IC), *R*^2^, and χ^2^, were performed to assess the association among nodules presence/absence and cytology categories (TIR2, TIR3a, TIR3b vs. TIR4, and TIR5) with categorical variables included in this study. To the bivariate proportional OR model performed to assess the association among the nodules presence/absence and cytology categories (TIR2, TIR3a, TIR3b vs. TIR4/TIR5) with continuous variables included in this study (*p*-value, 95% CI, and *R*^2^). In addition, two multiple linear regression analysis models (stepwise method), expressed as *R*^2^, Beta (β) and *t*, with the nodules presence/absence as a dependent variable were used to estimate the predictive value of: sex, lifestyle characteristics, BMI categories, and PREDIMED scores (Model 1), and each item included in PREDIMED questionnaire and PREDIMED score (Model 2). Further two multiple linear regression analysis models (stepwise method), expressed as *R*^2^, Beta (β), and *t*, with the cytology categories (TIR2, TIR3a, TIR3b vs. TIR4/TIR5) as a dependent variable were used to estimate the predictive value of: sex, lifestyle characteristics, BMI categories, and PREDIMED scores (Model 1), and each item included in PREDIMED questionnaire and PREDIMED score (Model 2). Receiver operator characteristic (ROC) curves analysis were performed to determine area under the curve (AUC), criterion, sensitivity and specificity, standard error, and 95% IC as well as cut-off values for PREDIMED score in detecting presence of thyroid nodules and the high-risk of malignancy (TIR4/TIR5). Variables with a variance inflation factor (VIF) >10 were excluded to avoid multicollinearity. The *p*-values below 5% were considered statistically significant. Data were analyzed using the IBM SPSS Statistics Software (PASW Version 21.0, SPSS Inc., Chicago, IL, USA) and MedCalc® package (Version 12.3.0 1993–2012 MedCalc Software bvba—MedCalc Software, Mariakerke, Belgium).

## Results

The study population consisted of 794 subjects, 240 males (30.2%) and 554 females (69.8%), aged 18–65 years, with BMIs ranging from 19.4 to 55.3 kg/m^2^. There were 215 current smokers (27.1%), while there were 416 physically active subject (52.4%). Regarding BMI classes, the most common was grade III obesity (170 patients, 21.4%). In addition, based on PREDIMED categories, the majority of subjects had an average adherence to the MD (49.4%), while only 15.7% of the study population showed a high adherence to the MD. Thyroid nodular disease were present in 391 (49.2%) participants, with a clear gender difference in thyroid nodular disease prevalence (χ^2^ = 90.06; *p* < 0.001). The descriptive characteristics, including gender, age, lifestyle characteristics, anthropometric measurements, nutritional, and thyroid parameters of the study population are given in Table 1.

**Table 1:** The descriptive characteristics, including gender, age, lifestyle characteristics, anthropometric measurements, nutritional, and thyroid parameters of the study population.

**Parameters**	**Mean ±SD or *n*, % *n*. 794**
Sex (Male)	240 (30.2%)
Age (Years)	43.13 ± 11.98
**Lifestyle characteristics**
Smoking (Yes)	215 (27.1%)
Physical activity (Yes)	416 (52.4%)
**Anthropometric measurements**	
Weight (kg)	93.33 ± 25.88
Height (m)	1.68 ± 0.09
BMI (kg/m^2^)	32.86 ± 7.94
Normal weight (*n*, %)	166 (20.9%)
Overweight (*n*, %)	161 (20.3%)
Grade I obesity (*n*, %)	157 (19.8%)
Grade II obesity (*n*, %)	140 (17.6%)
Grade III obesity (*n*, %)	170 (21.4%)
**Nutritional parameters**	
PREDIMED score	6.61 ± 2.86
Low adherence to the MD	277 (34.9%)
Average adherence to the MD	392 (49.4%)
High adherence to the MD	125 (15.7%)
**Thyroid nodules**	
Presence	391 (49.2%)
Absence	403 (50.8%)

*SD, Standard deviations; BMI, Body mass index; PREDIMED, Prevención con Dieta Mediterránea; MD, Mediterranean Diet*.

Table 2 showed the same characteristics reported in Table 1 of the study population according to presence/absence of thyroid nodular disease. In particular, there were no sex, age, and BMI differences in the two categories. Contrariwise, participants with thyroid nodular disease were more frequently smokers (*p* < 0.001) and practiced less physical activity (*p* < 0.001) than to those without thyroid nodular disease. In addition, patients with thyroid nodular disease had a lower adherence to the MD (*p* < 0.001) compared to those without thyroid nodular disease.

**Table 2:** The descriptive characteristics, including gender, age, lifestyle characteristics, anthropometric measurements, nutritional, and thyroid parameters of the study population according to presence/absence of thyroid nodular disease.

**Parameters**	**Nodules** **presence** ***n*. 391** **(49.2%)**	**Nodules** **absence** ***n*. 403** **(50.8%)**	*p-value
**Sex**			
Males (*n*, %)	110 (28.1%)	130 (32.3%)	χ^2^ = 1.41, *p* = 0.235
Females (*n*, %)	281 (71.9%)	273 (67.7%)	
Age (Years)	42.83 ± 13.59	43.43 ± 10.19	0.480
**Lifestyle characteristics**			
Smoking (Yes)	155 (39.6)	60 (14.9%)	χ^2^ = 60.33, *p* < 0.001
Physical activity (Yes)	143 (36.6)	273 (67.7%)	χ^2^ = 76.05, *p* < 0.001
**Anthropometric measurements**			
BMI (kg/m^2^)	33.27 ± 8.08	32.45 ± 7.79	0.145
Normal weight (*n*, %)	70 (17.9%)	96 (23.8%)	χ^2^ = 3.85, *p* = 0.066
Overweight (*n*, %)	90 (23.0%)	71 (17.6%)	χ^2^ = 3.25, *p* = 0.071
Grade I obesity (*n*, %)	76 (19.4%)	81 (20.1%)	χ^2^ = 0.02, *p* = 0.884
Grade II obesity (*n*, %)	70 (17.9%)	70 (17.4%)	χ^2^ = 0.01, *p* = 0.917
Grade III obesity (*n*, %)	85 (21.7%)	85 (21.1%)	χ^2^ = 0.02, *p* = 0.892
**Nutritional parameters**			
PREDIMED score	5.27 ± 2.62	7.90 ± 2.44	<0.001
Low adherence to the MD	212 (54.2%)	65 (16.1%)	χ^2^ = 125.09, *p* < 0.001
Average adherence to the MD	158 (40.4%)	234 (58.1%)	χ^2^ = 24.05, *p* < 0.001
High adherence to the MD	21 (5.4%)	104 (25.8%)	χ^2^ = 60.95, *p* < 0.001

BMI, Body mass index; PREDIMED, Prevención con Dieta Mediterránea; MD, Mediterranean Diet. ^*^A significant difference (p < 0.05).

Analyzing the response frequency of items included in the PREDIMED questionnaire in detail, according to presence/absence of thyroid nodular disease, we found that the patients with thyroid nodular disease consumed less healthy foods of MD including extra-virgin olive oil (*p* < 0.001), vegetables (*p* < 0.001), and fish (*p* < 0.001), and more non-Mediterranean foods, including red/processed meats (*p* < 0.001) and butter (*p* = 0.004) as compared with the subjects without thyroid nodular disease; Table 3.

**Table 3:** Response frequency of dietary components included in the PREDIMED questionnaire according to presence/absence of thyroid nodular disease.

**Questions of PREDIMED questionnaire**	**Nodules presence**		**Nodules absence**			
	***n***	**%**	***n***	**%**	**χ^2^**	*p-value
Use of extra virgin olive oil as main culinary lipid	273	69.8	341	84.6	66.22	<0.001
Extra virgin olive oil >4 tablespoons	177	45.3	218	54.1	5.84	0.016
Vegetables ≥2 servings/day	130	33.2	247	61.3	61.46	<0.001
Fruits ≥3 servings/day	100	25.6	286	71.0	110.67	<0.001
Red/processed meats <1/day	129	33.0	305	75.7	144.22	<0.001
Butter, cream, margarine <1/day	234	59.8	200	49.6	7.96	0.004
Soda drinks <1/day	184	47.1	199	49.4	0.34	0.559
Wine glasses ≥7/week	95	24.3	162	40.2	22.20	<0.001
Legumes ≥3/week	146	37.3	228	56.6	28.70	<0.001
Fish/seafood ≥3/week	79	20.2	329	81.6	297.37	<0.001
Commercial sweets and confectionery ≤ 2/week	123	31.5	182	45.2	15.18	0.001
Tree nuts ≥3/week	89	22.8	151	37.5	19.66	<0.001
Poultry more than red meats	177	45.3	210	52.1	6.64	0.010
Use of sofrito sauce ≥2/week	121	30.9	161	40.0	3.45	0.063

PREDIMED, Prevención con Dieta Mediterránea. ^*^A significant difference (p < 0.05).

**Figure 1:**
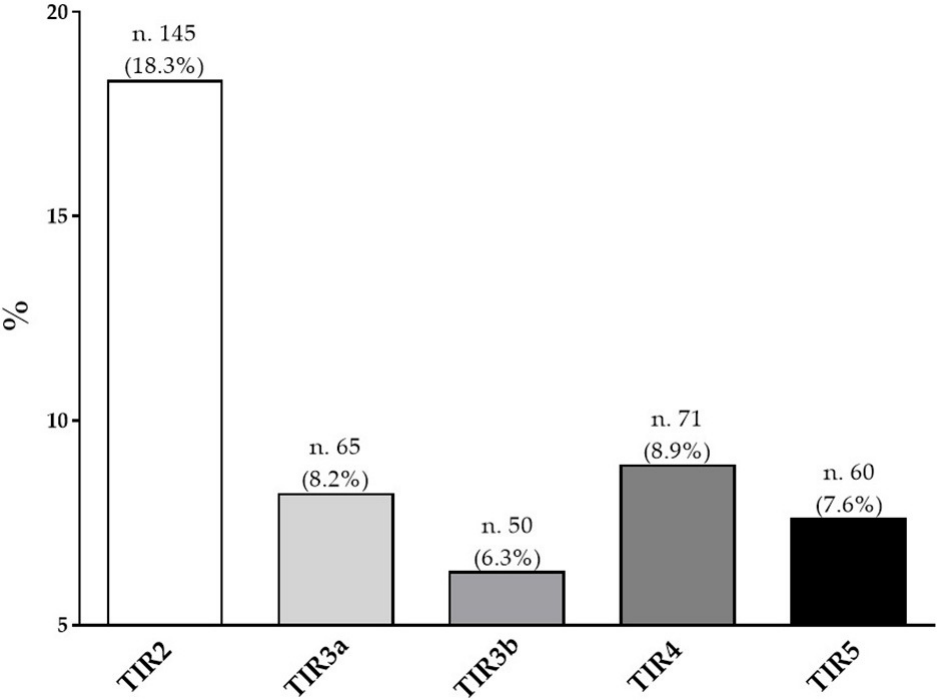
Percentage of the five diagnostic cytology categories based on 2013 Italian thyroid cytology classification system.

Figure 1 showed the percentage of the five diagnostic cytology categories based on 2013 Italian thyroid cytology classification system. Most of the participants presented a TIR2 (18.3%), followed by a TIR4 (8.9%).

Table 4 reported gender, age, lifestyle characteristics, anthropometric measurements, and nutritional parameters of the study population according to the five diagnostic cytology categories based on 2013 Italian thyroid cytology classification system. Patients with TIR5 were mostly male (*p* < 0.001) and were younger than subjects with TIR2 (*p* < 0.001). In addition, a higher percentage of smokers and sedentary were present in patients with TIR5 compared to the other TIR categories (*p* < 0.001). In addition, a higher percentage of patients with grade III obesity (41.7%) were present in patients with TIR5 compared to other TIRs (*p* < 0.001). With respect to nutritional parameters, patients with TIR 5 had the lowest adherence to the MD (2.15 ± 1.12 score) and had the highest percentage of the low adherence to this dietary pattern (98.3%) than other TIRs (*p* < 0.001; Table 4).

**Table 4:** Gender, age, lifestyle characteristics, anthropometric measurements, and nutritional parameters of the study population according to the five diagnostic cytology categories based on 2013 Italian thyroid cytology classification system.

**Parameters**	**TIR2 *n*. 145 (18.3%)**	**TIR3a *n*. 65 (8.2%)**	**TIR3b *n*. 50 (6.3%)**	**TIR4 *n*. 71** **(8.9%)**	**TIR5 *n*. 60** **(7.6%)**	*p-value
**Sex**						
Males (*n*, %)	26 (17.9%)	13 (20.0%)	14 (28.0%)	24 (33.8%)	33 (55.0%)	χ^2^ = 32.14, *p* < 0.001
Females (*n*, %)	119 (82.1%)	52 (80.0%)	36 (72.0%)	47 (66.2%)	27 (45.0%)	
Age (Years)	48.73 ± 11.29	44.69 ± 13.80	37.32 ± 11.98	37.25 ± 13.86	37.71 ± 13.31	<0.001
**Lifestyle characteristics**						
Smoking (Yes)	22 (15.2%)	17 (26.2%)	19 (38.0%)	40 (56.3%)	57 (95.0%)	χ^2^ = 126.40, *p* < 0.001
Physical activity (Yes)	81 (55.9%)	30 (46.2%)	21 (42.0%)	7 (9.9%)	4 (6.7%)	χ^2^ = 71.44, *p* < 0.001
**Anthropometric measurements**						
BMI (kg/m^2^)	27.80 ± 6.06	29.94 ± 7.33	39.64 ± 5.08	38.34 ± 5.44	38.77 ± 6.72	<0.001
Normal weight (*n*, %)	52 (35.9%)	15 (23.1%)	1 (2.0%)	0 (0.0%)	0 (0.0%)	χ^2^ = 71.36, *p* < 0.001
Overweight (*n*, %)	55 (37.9%)	22 (33.8%)	0 (0.0%)	8 (11.3%)	5 (8.3%)	χ^2^ = 56.18, *p* < 0.001
Grade I obesity (*n*, %)	20 (13.8%)	15 (23.1%)	12 (24.0%)	17 (23.9%)	12 (20.0%)	χ^2^ = 5.10, *p* = 0.278
Grade II obesity (*n*, %)	11 (7.6%)	7 (10.8%)	16 (32.0%)	20 (28.2%)	16 (26.7%)	χ^2^ = 27.74, *p* < 0.001
Grade III obesity (*n*, %)	7 (4.8%)	6 (9.2%)	21 (42.0%)	26 (36.6%)	25 (41.7%)	χ^2^ = 65.66, *p* < 0.001
**Nutritional parameters**						
PREDIMED score	7.49 ± 1.76	6.28 ± 2.02	4.04 ± 1.54	3.31 ± 1.10	2.15 ± 1.12	<0.001
Low adherence to the MD	21 (14.5%)	24 (36.9%)	38 (76.0%)	70 (98.6%)	59 (98.3%)	χ^2^ = 212.99, *p* < 0.001
Average adherence to the MD	105 (72.4%)	39 (60.0%)	12 (24.0%)	1 (1.4%)	1 (1.7%)	χ^2^ = 159.88, *p* < 0.001
High adherence to the MD	19 (13.1%)	2 (3.1%)	0 (0.0%)	0 (0.0%)	0 (0.0%)	χ^2^ = 28.01, *p* < 0.001

BMI, Body mass index; PREDIMED, Prevención con Dieta Mediterránea; MD, Mediterranean Diet. ^*^A significant difference (p < 0.05).

In Table 5 we reported the responses of each item included in PREDIMED questionnaire in the study population grouped according to the five diagnostic cytology categories based on 2013 Italian thyroid cytology classification system. As showed in the Table 5, the participants with TIR5 exhibited significant differences compared with other TIRs in terms of the consumption of all PREDIMED items, except for poultry meat (*p* = 0.096).

**Table 5:** Responses of each item included in PREDIMED questionnaire in the study population grouped according to the five diagnostic cytology categories based on 2013 Italian thyroid cytology classification system.

**Questions of PREDIMED questionnaire**	**TIR2** ***n*****. 145** **(18.3%)**		**TIR3a** ***n*****. 65** **(8.2%)**		**TIR3b** ***n*****. 50** **(6.3%)**		**TIR4** ***n*****. 71** **(8.9%)**		**TIR5** ***n*****. 60** **(7.6%)**			
	***n***	**%**	***n***	**%**	***n***	**%**	***n***	**%**	***n***	**%**	**χ^2^**	*p-value
Use of extra virgin olive oil as main culinary lipid	136	93.8	58	89.2	29	58.0	30	42.3	20	33.3	117.99	<0.001
Extra virgin olive oil >4 tablespoons	95	65.5	38	58.5	17	34.0	19	26.8	8	13.3	65.64	<0.001
Vegetables ≥2 servings/day	73	50.3	24	36.9	16	32.0	13	18.3	4	6.7	45.77	<0.001
Fruits ≥3 servings/day	47	32.4	20	30.8	17	34.0	15	21.1	1	1.7	25.11	<0.001
Red/processed meats <1/day	92	63.4	14	24.5	16	32.0	7	9.9	0	0.0	111.47	<0.001
Butter, cream, margarine <1/day	122	84.1	55	84.6	13	26.0	21	29.6	23	38.3	114.66	<0.001
Soda drinks <1/day	113	77.9	36	55.4	17	34.0	15	21.1	3	5.0	126.56	<0.001
Wine glasses ≥7/week	24	16.6	24	36.9	11	22.0	21	29.6	15	25.0	11.60	0.021
Legumes ≥3/week	76	52.4	34	52.3	13	26.0	17	23.9	6	10.0	47.67	<0.001
Fish/seafood ≥3/week	59	40.7	6	9.2	6	12.0	8	11.3	0	0.0	63.39	<0.001
Commercial sweets and confectionery ≤ 2/week	59	40.7	26	40.0	18	36.0	13	18.3	7	11.7	25.00	0.001
Tree nuts ≥3/week	63	43.4	10	15.4	5	10.0	9	12.7	2	3.3	58.93	<0.001
Poultry more than red meats	36	24.8	22	33.8	12	24.0	27	38.0	24	40.0	7.89	0.096
Use of sofrito sauce ≥2/week	88	60.7	41	63.1	12	24.0	20	28.2	16	26.7	48.13	<0.001

PREDIMED, Prevención con Dieta Mediterránea. ^*^ A significant difference (p < 0.05).

Table 6 reported the results of a multinomial logistic regression to assess the association between the thyroid nodules presence/absence and categorical variables included in this study. The presence of thyroid nodular disease was significantly associated with all categorical variables (*p* < 0.001), except gender (*p* = 0.206) and grade I obesity (*p* = 0.815). In particular, the presence of thyroid nodular disease was associated with smoking (OR 3.76, *p* < 0.001), physically inactive subjects (OR 0.28, *p* < 0.001), the BMI classes, and the lowest adherence to the MD (OR 6.16, *p* < 0.001).

**Table 6:** The results of a multinomial logistic regression to assess the association between the thyroid nodules presence/absence and categorical variables included in this study.

**Parameters**	**OR**	*p-value	**95% CI**	***R^**2**^***	**χ^2^**
**Sex**					
Males/Females	1.22	0.206	0.90–1.65	0.002	1.60
**Lifestyle characteristics**					
Smoking	3.76	<0.001	2.67–5.28	0.076	63.17
Physical activity	0.28	<0.001	0.21–0.37	0.094	78.59
**Anthropometric measurements**					
Normal weight	0.69	0.041	0.49–0.99	0.005	4.22
Overweight	1.40	0.050	0.98–1.98	0.005	3.59
Grade I obesity	0.96	0.815	0.67–1.36	0.001	0.55
Grade II obesity	0.66	0.018	0.46–0.93	0.007	5.66
Grade III obesity	1.76	0.003	1.21–2.55	0.011	8.99
**Nutritional parameters**					
Low adherence to the MD	6.16	<0.001	4.42–8.58	0.153	131.68
Average adherence to the MD	0.49	<0.001	0.37–0.65	0.031	24.88
High adherence to the MD	0.16	<0.001	0.10–0.27	0.081	67.48

MD, Mediterranean diet; OR, odds ratio; CI, confidence interval. *A significant difference (p < 0.05).

To the bivariate proportional OR model performed to assess the association between the thyroid nodules presence/absence and continuous variables, included age (OR = 0.99, *p* = 0.480, 95% CI = 0.98–1.01, *R*^2^ = 0.001), BMI (OR = 1.01, *p* = 0.145, 95% CI = 0.99–1.03, *R*^2^ = 0.003), and PREDIMED score (OR = 0.67, *p* < 0.001, 95% CI = 0.63–0.72, *R*^2^ = 0.208).

Table 7 summarized the results of a multinomial logistic regression to assess the association between the cytology categories (TIR2, TIR3a, TIR3b vs. TIR4/TIR5) and categorical variables included in this study. The TIR4/TIR5 category was significantly associated with all categorical variables (*p* < 0.001), except grade I obesity (OR 1.29, *p* = 0.339). In particular, the TIR4/TIR5 category were associated with smoking (OR 9.94, *p* < 0.001), physically inactive subjects (OR 0.09, *p* < 0.001), the BMI classes, and the lowest adherence to the MD (OR 137.55, *p* < 0.001; Table 7).

**Table 7:** Results of a multinomial logistic regression to assess the association between the cytology categories (TIR2, TIR3a, TIR3b vs. TIR4/TIR5) and categorical variables included in this study.

**Parameters**	**OR**	*p-value	**95% CI**	***R^**2**^***	**χ^2^**
**Sex**					
Males/Females	0.33	<0.001	0.21–0.53	0.55	22.32
**Lifestyle characteristics**					
Smoking	9.94	<0.001	6.10–16.18	0.224	99.12
Physical activity	0.09	<0.001	0.05–0.17	0.180	77.57
**Anthropometric measurements**					
Normal weight	0.04	<0.001	0.01–0.18	0.115	47.95
Overweight	0.26	<0.001	0.14–0.49	0.053	21.22
Grade I obesity	1.29	0.339	0.77–2.17	0.002	0.90
Grade II obesity	2.52	0.001	1.49–4.26	0.030	11.74
Grade III obesity	4.24	<0.001	2.56–7.01	0.080	32.63
**Nutritional parameters**					
Low adherence to the MD	137.55	<0.001	33.22–269.47	0.389	192.89
Average adherence to the MD	0.01	<0.001	0.01–0.043	0.331	156.90
High adherence to the MD	0.25	<0.001	0.18–0.51	0.044	17.73

MD, Mediterranean diet; OR, odds ratio; CI, confidence interval. *A significant difference (p < 0.05).

To the bivariate proportional OR model performed to assess the association between the cytology categories (TIR2, TIR3a, TIR3b vs. TIR4/TIR5) and continuous variables, included age (OR = 0.96, *p* < 0.001, 95% CI = 0.94–0.97, *R*^2^ = 0.076), BMI (OR = 1.15, *p* < 0.001, 95% CI = 1.12–1.19, *R*^2^ = 0.021), PREDIMED score (OR = 0.33, *p* < 0.001, 95% CI = 0.26–0.41, *R*^2^ = 0.462).

Table 8 showed the bivariate proportional OR model performed to assess the association between the nodules presence/absence and the responses of each item included in PREDIMED questionnaire. In particular, the presence of thyroid nodules was associated with all items included in PREDIMED questionnaire, except for soda drinks (*p* = 0.513) and use of sofrito sauce (*p* = 0.054; Table 8).

**Table 8:** The bivariate proportional OR model performed to assess the association between the thyroid nodule presence/absence and the responses of each item included in PREDIMED questionnaire.

**Questions of PREDIMED questionnaire**	**OR**	*p-value	**95% CI**	***R^**2**^***	**χ^2^**
Use of extra virgin olive oil as main culinary lipid	0.42	<0.001	0.29–0.59	0.031	25.08
Extra virgin olive oil >4 tablespoons	0.70	0.013	0.53–0.93	0.008	6.19
Vegetables ≥2 servings/day	0.32	<0.001	0.24–0.42	0.077	63.46
Fruits ≥3 servings/day	0.14	<0.001	0.10–0.19	0.193	169.92
Red/processed meats <1/day	0.16	<0.001	0.11–0.22	0.173	150.83
Butter, cream, margarine <1/day	1.51	0.004	0.14–2.00	0.010	8.38
Soda drinks <1/day	0.91	0.513	0.69–1.20	0.001	0.43
Wine glasses ≥7/week	0.48	<0.001	0.35–0.65	0.029	23.13
Legumes ≥3/week	0.46	<0.001	0.34–0.61	0.037	29.67
Fish/seafood ≥3/week	0.06	<0.001	0.04–0.08	0.334	322.25
Commercial sweets and confectionery ≤ 2/week	0.56	<0.001	0.42–0.75	0.020	15.83
Tree nuts ≥3/week	0.49	<0.001	0.36–0.67	0.026	20.54
Poultry more than red meats	0.67	0.008	0.50–0.90	0.009	7.04
Use of sofrito sauce ≥2/week	0.76	0.054	0.58–1.00	0.005	3.72

PREDIMED, Prevención con Dieta Mediterránea; OR, odds ratio; CI, confidence interval. *A significant difference (p < 0.05).

Table 9 reported the bivariate proportional OR model performed to assess the association between the cytology categories (TIR2, TIR3a, TIR3b vs. TIR4/TIR5) and the responses of each item included in PREDIMED questionnaire. TIR4/TIR5 category was associated with all items included in PREDIMED questionnaire, except for wine glasses ≥7/week (*p* = 0.298; Table 9).

**Table 9:** The bivariate proportional OR model performed to assess the association between the cytology categories (TIR2, TIR3a, TIR3b vs. TIR4/TIR5) and the responses of each item included in PREDIMED questionnaire.

**Questions of PREDIMED questionnaire**	**OR**	*p-value	**95% CI**	***R^**2**^***	**χ^2^**
Use of extra virgin olive oil as main culinary lipid	0.10	<0.001	0.06–0.17	0.210	91.93
Extra virgin olive oil >4 tablespoons	0.19	<0.001	0.12–0.31	0.122	50.98
Vegetables ≥2 servings/day	0.20	<0.001	0.11–0.34	0.098	40.19
Fruits ≥3 servings/day	0.29	<0.001	0.16–0.52	0.050	20.21
Red/processed meats <1/day	0.06	<0.001	0.03–0.14	0.189	81.81
Butter, cream, margarine <1/day	0.18	<0.001	0.12–0.29	0.135	56.66
Soda drinks <1/day	0.09	<0.001	0.05–0.16	0.217	95.59
Wine glasses ≥7/week	1.29	0.298	0.79–2.09	0.003	1.07
Legumes ≥3/week	0.24	<0.001	0.14–0.39	0.086	35.29
Fish/seafood ≥3/week	0.17	<0.001	0.08–0.37	0.070	28.42
Commercial sweets and confectionery ≤ 2/week	0.28	<0.001	0.16–0.47	0.064	25.87
Tree nuts ≥3/week	0.21	<0.001	0.011–0.42	0.065	26.26
Poultry more than red meats	1.73	0.016	0.11–2.70	0.015	5.77
Use of sofrito sauce ≥2/week	0.32	<0.001	0.20–0.50	0.064	25.91

PREDIMED, Prevención con Dieta Mediterránea; OR, odds ratio; CI, confidence interval. *A significant difference (p < 0.05).

To compare the relative predictive power of sex, smoking, physical activity, BMI categories, PREDIMED score, and each item included in PREDIMED questionnaire associated with thyroid nodules presence/absence, we performed two multiple regression analysis. The first model included sex, lifestyle characteristics, BMI categories, and PREDIMED scores. In this model, PREDIMED score was entered at the first step (*p* < 0.001), followed by the BMI (*p* < 0.001). Results were reported in Table 10. The second model included each item included in PREDIMED questionnaire and PREDIMED score. In this model, fish consumed was entered at the first step (*p* < 0.001).

**Table 10:** Multiple regression analysis models (stepwise method) with the thyroid nodules presence/absence as a dependent variable to estimate the predictive value of sex, lifestyle characteristics, BMI categories, PREDIMED scores, and each item included in PREDIMED questionnaire.

**Parameters**	**Multiple regression analysis**			
	***R^**2**^***	**β**	**t**	*p-value
**Model 1**				
PREDIMED score	0.211	−0.461	−14.61	<0.001
BMI categories	0.287	−0.334	−9.24	<0.001
**Model 2**				
Fish/seafood ≥3/week	0.377	−0.614	−21.92	<0.001
Fruits ≥3 servings/day	0.431	−0.255	−8.77	<0.001
Red/processed meats <1/day	0.455	−0.173	−7.43	<0.001
PREDIMED score	0.462	−0.094	−3.54	<0.001

PREDIMED, Prevención con Dieta Mediterránea; BMI, Body mass index. ^*^A significant difference (p < 0.05).

To compare the relative predictive power of sex, smoking, physical activity, BMI categories, PREDIMED score, and each item included in PREDIMED questionnaire associated with the cytology categories (TIR2, TIR3a, TIR3b vs. TIR4/TIR5), we performed two multiple regression analysis. The first model included sex, lifestyle characteristics, BMI categories, and PREDIMED scores. In this model, PREDIMED score was entered at the first step (*p* < 0.001), followed by the smoking (*p* < 0.001). Results were reported in Table 11. The second model included each item included in PREDIMED questionnaire and PREDIMED score. In this model, PREDIMED score consumed was entered at the first step (*p* < 0.001; Table 11).

**Table 11:** Multiple regression analysis models (stepwise method) with the cytology categories (TIR2, TIR3a, TIR3b vs. TIR4/TIR5) as a dependent variable to estimate the predictive value of sex, lifestyle characteristics, BMI categories, PREDIMED scores, and each item included in PREDIMED questionnaire.

**Parameters**	**Multiple regression analysis**			
	***R^**2**^***	**β**	**t**	*p-value
**Model 1**				
PREDIMED score	0.626	−0.792	−25.55	<0.001
Smoking	0.664	0.224	6.70	<0.001
BMI categories	0.669	0.104	2.63	0.009
**Model 2**				
PREDIMED score	0.626	−0.792	−25.55	<0.001
Use of extra virgin olive oil as main culinary lipid	0.639	−0.144	−4.24	<0.001
Red/processed meats <1/day	0.653	−0.142	−4.12	<0.001

PREDIMED, Prevención con Dieta Mediterránea; BMI, Body mass index. ^*^A significant difference (p < 0.05).

**Figure 2:**
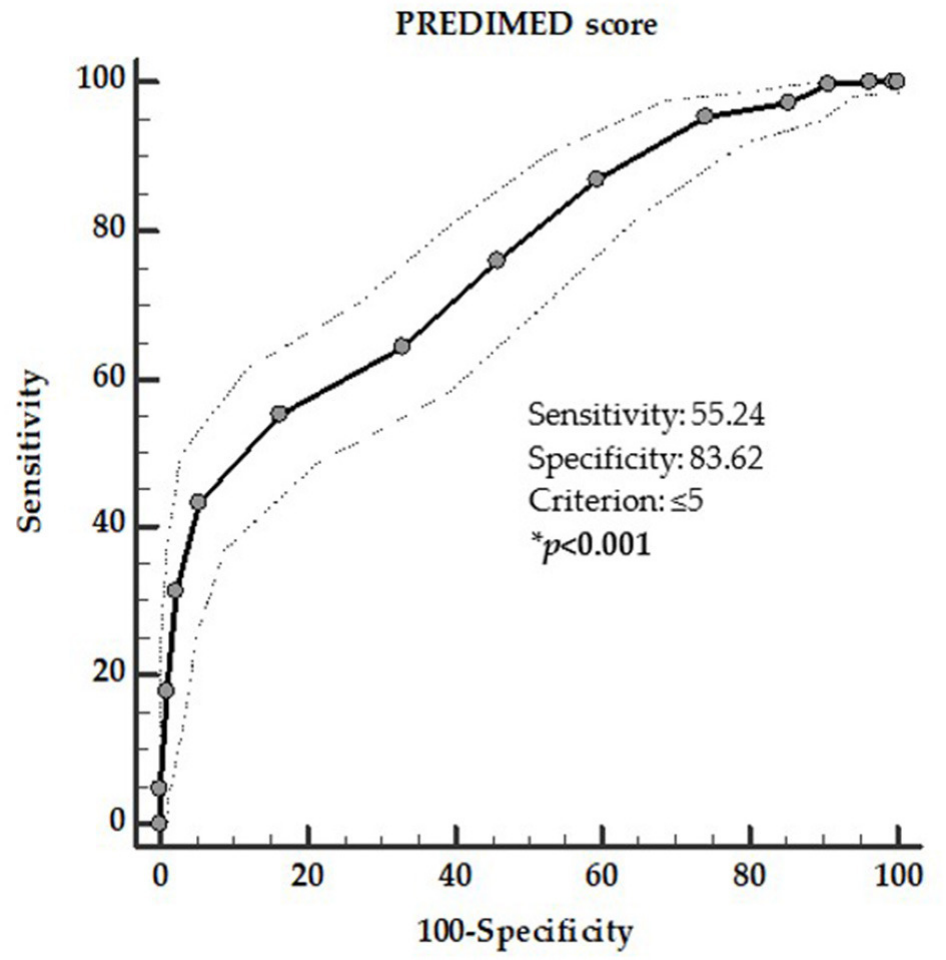
ROC for predictive values of PREDIMED score in detecting the presence of thyroid nodules. A PREDIMED score ≤ 5 (*p* = 0.001, AUC 0.758, standard error 0.017, 95% CI 0.725–0.791) predicted the presence of thyroid nodules. *A significant difference (*p* < 0.05).

To determine the cut-off values of PREDIMED score that were predictive of the presence of thyroid nodules and TIR4/TIR5 cytology category, two ROC analysis were performed. In the first ROC analysis the threshold values of adherence to the MD predicting the presence of thyroid nodules were found at PREDIMED score ≤ 5 (*p* = 0.001, AUC 0.758, standard error 0.017, 95% CI = 0.725–0.791; Figure 2).

**Figure 3:**
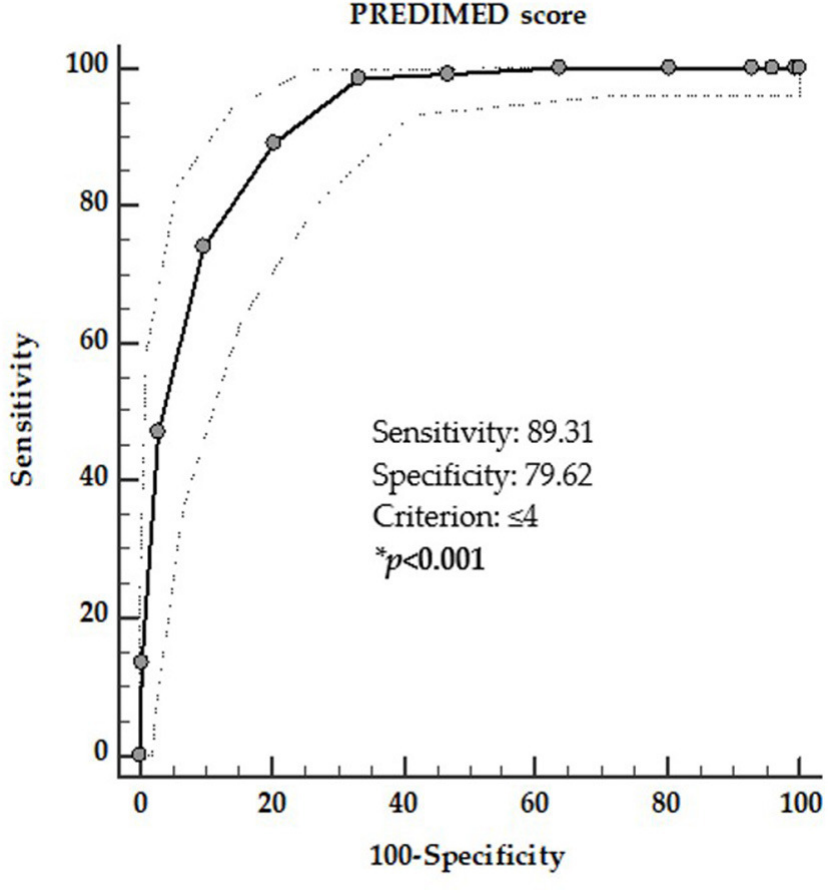
ROC for predictive values of PREDIMED score in detecting the presence of TIR4/TIR5 cytology category. A PREDIMED score ≤ 4 (*p* = 0.001, AUC 0.921, standard error 0.013, 95% CI 0.896–0.947) predicted the presence of TIR4/TIR5 cytology category. *A significant difference (*p* < 0.05).

In the second ROC analysis the threshold values of adherence to the MD that was predictive of the presence of TIR4/TIR5 cytology category was found at PREDIMED score ≤ 4 (*p* = 0.001, AUC 0.921, standard error 0.013, 95% CI = 0.896–0.947; Figure 3).

## Discussion

In this study, we evaluated the association of adherence to the MD with the thyroid nodular disease and the cytological classification in a large cohort of adult population. As novel finding, we reported that adherence to the MD was reduced in patients with thyroid nodular disease and in particular in those with cytological category with high-risk of malignancy, independently of BMI.

### Thyroid Nodular Disease and Mediterranean Diet

A PREDMED score ≤ 5 was reported in more than 50% of patients with thyroid nodules vs. 16% of those without thyroid nodules. Analyzing the single items of the PREDIMED questionnaire, it was evident that the consumption of almost all the Mediterranean healthy foods was lower in patients with thyroid nodules. At multiple regression analysis, the low consumption of fish and fruits, and the high consumption of red meats were associated with the presence of thyroid nodules. At regression analysis, the low adherence to the MD entered before than BMI as the best predictor of the presence of thyroid nodules. In the context of the accurate clinical evaluation of thyroid nodular disease, the PREDIMED score ≤ 5 could be served as an adjunctive predictor for helping in characterizing patients with presence of thyroid nodules.

Previous studies have mainly focused on the correlation between adherence to the MD and reduced risk of several cancer types and mortality (47). In addition, several studies have been carried out on the intake of single nutrients and foods and the risk of thyroid cancer, summarized in a recent narrative review (1). In particular, evidence reported that many foods typical of the MD, including fish and iodine-rich foods, fruits and vegetables, might exert protective effects on the reduction of thyroid cancer risk. Conversely, alcohol intake (48, 49) and red meat consumption (50) exert negative effects on thyroid cancer risk. Indeed, it is well-known that an approach based on studying a single food does not take into account the synergistic and/or antagonistic interactions existing among nutrients and foods, and it probably has a suboptimal statistical power to assess associations with the risk of cancer (51). Yet, no study to date has attempted to investigate the association of adherence to the MD or single foods of MD and thyroid nodular disease.

Previous evidence investigated the association between obesity and thyroid nodular disease, indicating that patients with obesity were more likely to have thyroid nodules disease compared to normal weight individuals (6, 52). In particular, obesity has been linked to higher serum thyroid stimulating hormone (TSH) concentrations (53, 54), which consequently leads to higher thyroid volume (55) being the TSH a stimulating factor potentially contributing to the development of thyroid nodular disease and thyroid cancer (56).

Of interest, as found by Panagiotou et al. the nodule size was associated also with body fat percentage (57). In our study, we did not measure body composition; nevertheless, we found that patients with obesity had a higher prevalence of thyroid nodules compared with normal weight individuals. Studies showed that hyperglycaemia, insulin resistance and type 2 diabetes conditions that often coexist in patients with obesity, and represent risk factors for thyroid cancer independent of obesity. The proposal mechanism for this association was the activation of insulin and insulin-like growth factor pathway, which share affinity with insulin receptor and that could be over activated by compensatory hyperinsulinemia to insulin resistance (58). In addition, considering that the body size affects the iodine requirements, obesity is also considered one condition of relative iodine deficiency, which could predispose to thyroid nodular disease (59).

### Thyroid Nodule Cytology and Mediterranean Diet

The management of thyroid nodular disease is primarily based on morphologic classification of cytological samples obtained by FNA, complemented by clinical history of the nodule, imaging findings, and molecular markers test results (60). Nevertheless, ~25% of all FNAs falls into the indeterminate category, possibly resulting in unnecessary thyroid surgery (61). To increase the characterization of the cytological category with high-risk of malignancy by including the evaluation of modifiable risk factors, such as unhealthy foods and dietary patterns, could add an additional criterion to help the clinicians in the management of thyroid nodular disease. To our knowledge, this is the first study investigating the association between adherence to the MD according to the cytological classification of thyroid nodular disease in the subset of the study participants who have undergone FNA.

According to the five diagnostic cytology categories based on 2013 Italian thyroid cytology classification system, more than 18% of the study participants presented TIR2, while ~9 and 8% of the participants had high-risk categories, TIR4 and TIR5, respectively. The high-risk category (TIR4/TIR5) increased along the BMI categories. In particular, in class II obesity 54.9% of patients had high-risk categories (TIR4 28.2% and TIR5 26.7%), while 7.6% had TIR2; in class III obesity 78.3% of patients had high-risk categories (TIR4 36.6% and TIR5 41.7%), while 4.8% had TIR2. Consistently, the high-risk categories increased along with the reduction to adherence to the MD. In particular, up to 98% of patients with TIR5 had a low adherence to the MD.

Analyzing the single items of the PREDIMED questionnaire in relation with cytological categories, the consumption of almost all Mediterranean healthy foods was lower in patients with the high-risk category compared to patients with the low-risk category of malignancy. In particular, the low consumption of extra virgin olive oil, and the high consumption of red meats were associated with the high-risk category of malignancy. In addition, considering lifestyles (smoking and physical activity), BMI, and score of adherence to the MD, at multiple regression analysis, the PREDIMED score entered before smoking and BMI as predictor of high-risk category and a PREDIMED score ≤ 4 could help to characterize patients with high-risk category of malignancy.

Earlier evidence has mainly evaluated the association between single foods and differentiated thyroid cancer, nevertheless only few researches have been carried out studies to investigate the relationship between differentiated thyroid cancer and dietary patterns (21, 62–64).

In a case control study Sangsefidi et al. conducted among 309 individuals in northeast of Iran, authors investigated the association among four major dietary patterns including traditional dietary pattern, western dietary pattern, transitional dietary pattern, and healthy dietary pattern with the risk of having or developing thyroid cancer (64). The study results showed that only Western diet, characterized by high consumption of meat, eggs, refined cereals, cakes, sweet drinks, and alcohol intake, was associated with increased risk of thyroid cancer (64).

In contrast with results of Sangsefidi et al. (64) and Clero et al. (62) conducted in French Polynesia a case-control study in 229 cases of thyroid cancer and 371 controls, evaluating two different dietary patterns: Western diet and traditional Polynesian dietary pattern. The traditional Polynesian diet included specific foods of Pacific islands, including taro, cassava, tubers, sweet potato, fish and seafood, and fruits (as banana, mango, and pawpaw). Authors concluded that in French Polynesia there was no association between a Western diet and the risk of thyroid cancer. Conversely, the traditional Polynesian diet led to a weak reduced risk of thyroid cancer (62).

Results similar to ours were reported by Markaki et al. in a Greek population (63). This case-control study has been conducted in 113 patients with histologically verified thyroid cancer and 138 healthy controls, age, and sex-matched. The study results showed that dietary patterns characterized by the high consumption of fish, vegetables and fruits, led to a reduced risk for all thyroid cancers. In particular, high fish and vegetable intakes led to an increased risk of follicular thyroid cancer (63).

Moreover, in another study Liang J et al. in a case-control study of 390 historically confirmed incident thyroid cancer cases and 436 population controls conducted in Connecticut (2010–2011), examined the association between thyroid cancer risk and dietary pattern (21). The dietary patterns analyzed in this study were three: high protein and fat, starchy foods and desserts, and fruits and vegetables. The results of this study showed that the fruits and vegetables dietary pattern was significantly correlated with a reduced risk of overall thyroid cancer. Contrariwise, the starchy foods and desserts dietary pattern was positively associated with an overall thyroid cancer risk among men (21).

All the above-mentioned studies supported the link between specific foods characteristic of the MD and reduced thyroid cancer risk, although the exact mechanisms through which MD might affect thyroid cancer risk are still unclear. In particular, the low adherence to the MD is characterized by high consumption of red meat and low consumption of fish. Evidence indicated that processing of red meat or its cooking at a high temperature, is associated with the production of carcinogenic compounds, including polycyclic aromatic hydrocarbons, heterocyclic amines, and N-nitroso compounds. These compounds *via* increasing the proliferation of cells can promote carcinogenesis and result in increasing of overall cancer risk (65, 66). In this context, in 2002 Memon et al. showed a positive association between the high consumptions of mutton and lamb with thyroid cancer (67). Two very recent review summarized the role of individual foods on thyroid cancer risk concluding that to date, no definite association among dietary patterns, including MD and thyroid cancer, and its clinical severity and aggressiveness have been found (1, 68). Albeit the association between single foods and thyroid cancer is difficult to examine, the high consumption of vegetables, fruits, and fish foods, might exert protective effects on thyroid cancer risk. These foods, for the presence of essential nutrients including omega-3 polyunsaturated fatty acids, vitamins (retinol, vitamin D, and vitamin E), and minerals, have an anti-inflammatory properties and have been reported to be a protective factor in some types of tumors, including breast cancer (69, 70), neuroendocrine tumors (71, 72), and thyroid cancer (50, 73, 74).

Despite the attention that has been paid to the relation between diet and thyroid cancer risk, it is tempting to speculate that the beneficial anti-inflammatory effects of MD could be extended to the complex pathogenesis of thyroid nodular disease. However, although it has been reported that different foods and vitamins supplementation were differently associated with benign breast disease and diet, the relationship between adherence to the MD and thyroid nodular disease is an unexplored field, and associative clinical studies are still lacking (75). In particular, to the best of our knowledge the association between adherence to the MD and the cytological classification of thyroid nodular disease has not investigated previously.

Several studies supported the link between the low adherence to the MD and inflammation (31, 76). Indeed, cytokines, activating molecules in chronic inflammation, has been reported to facilitate the prevalence of thyroid nodular disease directly causing thyroiditis, playing a critical role in widely regulating cellular functions and promoting cellular proliferation, hyperplasia, differentiation, and survival (11). In this context, a recent study has been reported that the inflammation promotes the development of thyroid nodular disease, probably due to its indirect effect through inhibiting the synthesis of thyroid hormones, which results in the elevation of TSH levels (11). Furthermore, chronic inflammation is also associated with the malignant growth in thyroid nodular disease (77). In particular, the elevated TSH levels was implied of the development of thyroid nodular disease through the growth and proliferation of thyroid cell in different ways, thus leading to the formation to the goiter and thyroid nodular disease (11, 78). These results suggest that chronic inflammation, which coexists with the elevated TSH levels, in involved in the pathogenesis of thyroid nodular disease (11).

### Limitations

Despite these novel results, we are aware that our study presented several limitations. First, the observational design of this study does not allow any causal interpretation of the findings. In fact, the main limitation of this study is represented by the by the cross-sectional experimental design, which, although showing the association of the adherence to the MD with thyroid nodular disease and thyroid cancer malignancy, fails to provide any explanation of the causality of this association. Further, the study participants were not investigated by a medical history, including familiarity for thyroid nodular disease, exposure to radiation, and iodine intake. Second, data on thyroid function and additional ultrasound characteristics of thyroid nodular disease are lacking. Indeed, the relationship between adherence to the MD and thyroid function has been previously investigated in a cohort of subjects with overweight and obesity (79). In this cohort study, the Authors demonstrated that the higher adherence to the MD was independently associated to a slightly reduced thyroid function, and attributed this due to increase in the tissue and organ sensitivity to thyroid hormones and the reduction of thyroid hormones could be associated with the reduced formation of thyroid nodular disease (79). Third, we did not analyse inflammatory markers. Nevertheless, on the one side the anti-inflammatory and antioxidant effects of the MD is well-known (31, 80); on the other side it has reported that chronic inflammation promotes the development of thyroid nodular disease, probably due to its indirect effect *via* inhibiting the synthesis of thyroid hormone, which results in the elevation of TSH (11). A further important limitation in this study is that cytological data were not confirmed by histopathology. Finally, the proposed cut-off points of PREDIMED score for helping in characterizing patients with presence of thyroid nodules and cytological categories with high risk of malignancy (TIR4 and TIR5) should be validated in different population samples by further clinical trials.

### Strengths

This study has several strengths. The single-center study design of this research, although it could represent a selection bias due to the limits the generalizability of our findings, allowed us to increase the homogeneity of the sample. In particular, all participants included were came from the same geographical area, thus possibly sharing overall similar food availability. Moreover, we adjusted our data for a wide range of confounding factors that might have influence on thyroid nodular disease and thyroid cancer, including sex, age, physical activity, smoking, and BMI. The questionnaire of adherence to the MD used in this study, PREDIMED questionnaire was recently validated in different Mediterranean countries, including Italy (81). This questionnaire was not self-reported, but face-to-face administered by a certified Nutritionist, to reduce any bias related to the filling in of this questionnaire. Furthermore, the results of nutritional research using overall dietary patterns, such as MD compared to the study of the single food or nutrient is more amenable to translation into clinical practice (51, 82).

## Conclusions

**Figure 4:**
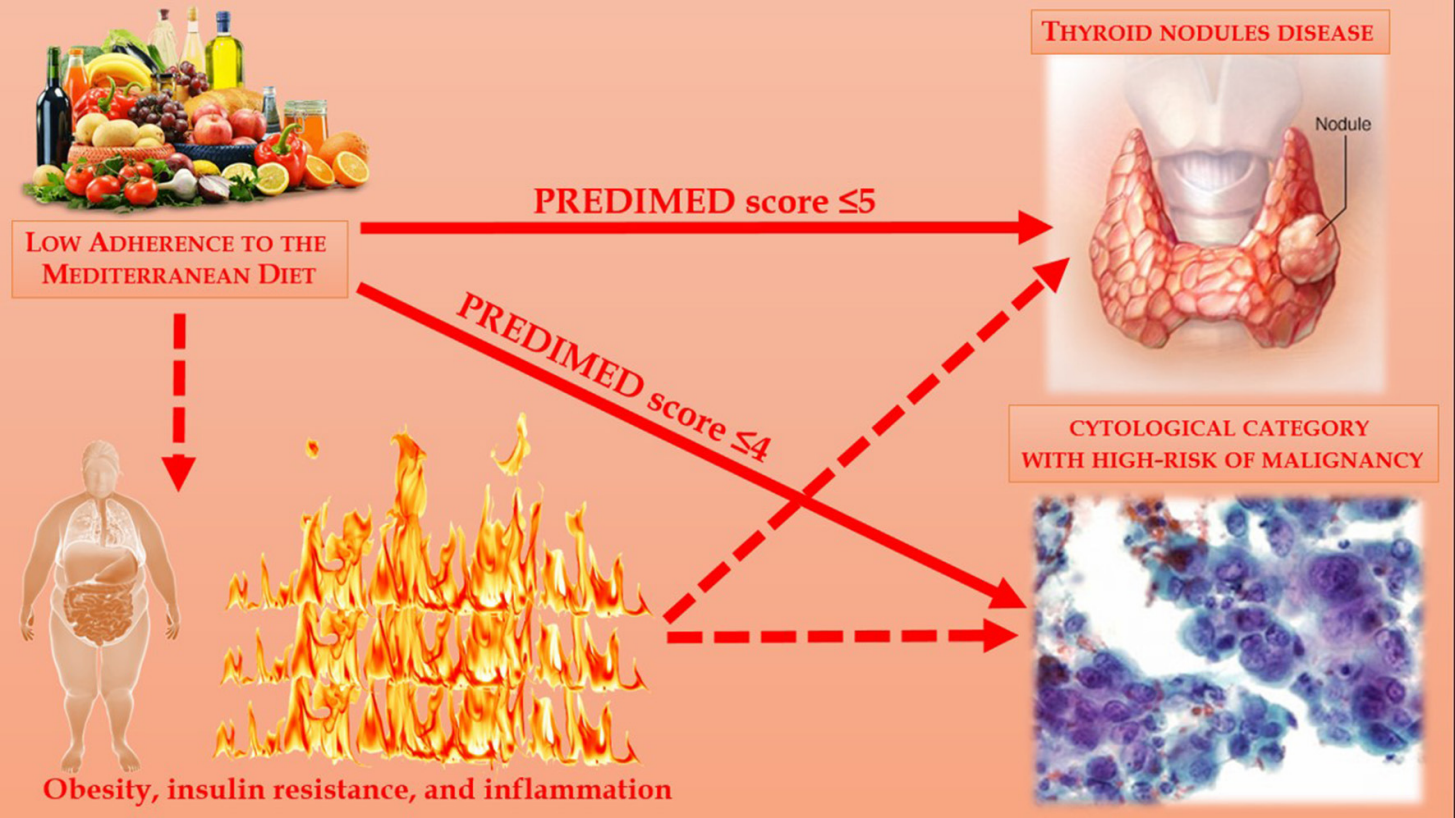
Association between adherence to the MD and thyroid nodular disease and cytological category with high-risk of malignancy were carried out.

Thyroid nodular disease is common in general population. To the best of our knowledge, no study to date that reported the association of adherence to the MD with thyroid nodular disease and cytological category with high-risk of malignancy were carried out. Even if the mechanisms underlying these observed associations remain largely speculative, the evaluation of adherence to the MD could represent an adjunctive tool in helping clinicians to better characterize patients with thyroid nodular disease and with cytological category with high-risk of malignancy. In this context, there is a strong rationale to support the inclusion of the evaluation to adherence to the MD into disease-specific dietary guidelines for the management of thyroid nodular disease, in order to reduce the inflammation that paves the way for insulin resistance and, consequently, the increased risk of developing thyroid nodular disease with high-risk category of malignancy; Figure 4. Further clinical studies on the associations between the prevalence of nodular thyroid disease and potential lifestyle characteristics, as dietary pattern including the MD, are warranted to confirm our observations.

## Data Availability Statement

The raw data supporting the conclusions of this article will be made available by the authors, without undue reservation.

## Ethics Statement

The studies involving human participants were reviewed and approved by Università Federico II di Napoli. The patients/participants provided their written informed consent to participate in this study.

## Author Contributions

LB and SS: conceptualization. LB, GM, FF, and GT: methodology. CV: formal analysis. GM, TP, LV, and SA: investigation. GA: data curation. LB and GA: writing—original draft preparation. LB, GA, and GM: writing—review and editing. AC and SS: visualization and supervision. All authors have read and agreed to the published version of the manuscript.

## Funding

Part of the research and open access of this article were supported with funds from the Ministry of Education, University and Research (PRIN. 2017N8CK4K).

## Conflict of Interest

The authors declare that the research was conducted in the absence of any commercial or financial relationships that could be construed as a potential conflict of interest.

## Publisher's Note

All claims expressed in this article are solely those of the authors and do not necessarily represent those of their affiliated organizations, or those of the publisher, the editors and the reviewers. Any product that may be evaluated in this article, or claim that may be made by its manufacturer, is not guaranteed or endorsed by the publisher.
